# CCR6^+^ Th cell populations distinguish ACPA positive from ACPA negative rheumatoid arthritis

**DOI:** 10.1186/s13075-015-0800-5

**Published:** 2015-11-30

**Authors:** Sandra M. J. Paulissen, Jan Piet van Hamburg, Nadine Davelaar, Heleen Vroman, Johanna M. W. Hazes, Pascal H. P. de Jong, Erik Lubberts

**Affiliations:** Departments of Rheumatology and Immunology, Erasmus MC, University Medical Center, Rotterdam, P.O. Box 2040, 3000 CA Rotterdam, The Netherlands; Department of Pulmonary Medicine, Erasmus MC, University Medical Center, Rotterdam, The Netherlands; Department of Rheumatology, Erasmus MC, University Medical Center, Rotterdam, The Netherlands

**Keywords:** ACPA, Rheumatoid arthritis, T cells, CCR6, Th17

## Abstract

**Introduction:**

Patients with rheumatoid arthritis (RA) can be separated into two major subpopulations based on the absence or presence of serum anti-citrullinated protein antibodies (ACPAs). The more severe disease course in ACPA^+^ RA and differences in treatment outcome between these subpopulations suggest that ACPA^+^ and ACPA^−^ RA are different disease subsets. The identification of T-helper (Th) cells specifically recognizing citrullinated peptides, combined with the strong association between HLA-DRB1 and ACPA positivity, point toward a pathogenic role of Th cells in ACPA^+^ RA. In this context we recently identified a potential pathogenic role for CCR6^+^ Th cells in RA. Therefore, we examined whether Th cell population distributions differ by ACPA status.

**Methods:**

We performed a nested matched case–control study including 27 ACPA^+^ and 27 ACPA^−^ treatment-naive early RA patients matched for disease activity score in 44 joints, presence of rheumatoid factor, sex, age, duration of complaints and presence of erosions. CD4^+^CD45RO^+^ (memory) Th cell distribution profiles from these patients were generated based on differential chemokine receptor expression and related with disease duration.

**Results:**

ACPA status was not related to differences in total CD4^+^ T cell or memory Th cell proportions. However, ACPA^+^ patients had significantly higher proportions of Th cells expressing the chemokine receptors CCR6 and CXCR3. Similar proportions of CCR4^+^ and CCR10^+^ Th cells were found. Within the CCR6^+^ cell population, four Th subpopulations were distinguished based on differential chemokine receptor expression: Th17 (CCR4^+^CCR10^−^), Th17.1 (CXCR3^+^), Th22 (CCR4^+^CCR10^+^) and CCR4/CXCR3 double-positive (DP) cells. In particular, higher proportions of Th22 (p = 0.02), Th17.1 (p = 0.03) and CCR4/CXCR3 DP (p = 0.01) cells were present in ACPA^+^ patients. In contrast, ACPA status was not associated with differences in Th1 (CCR6^−^CXCR3^+^; p = 0.90), Th2 (CCR6^−^CCR4^+^; p = 0.27) and T-regulatory (CD25^hi^FOXP3^+^; p = 0.06) cell proportions. Interestingly, CCR6^+^ Th cells were inversely correlated with disease duration in ACPA^−^ patients (R^2^ = −0.35; p < 0.01) but not in ACPA^+^ (R^2^ < 0.01; p = 0.94) patients.

**Conclusions:**

These findings demonstrate that increased peripheral blood CCR6^+^ Th cells proportions distinguish ACPA^+^ RA from ACPA^−^ RA. This suggests that CCR6^+^ Th cells are involved in the differences in disease severity and treatment outcome between ACPA^+^ and ACPA^−^ RA.

## Introduction

Rheumatoid arthritis (RA) is an autoimmune disease characterized by chronic synovial joint inflammation and auto-antibody presence [1, 2]. The presence of serum anti-citrullinated protein antibodies (ACPAs) is highly specific for RA, and ~70 % of patients with RA are ACPA^+^ [3–5]. Moreover, ACPAs are a useful marker for RA diagnosis as they can be present several years before clinical onset [6, 7].

The disease course in ACPA^+^ patients is worse than in ACPA^−^ patients, as shown, for example, from more development of erosions; and treatment outcomes differ between these groups [8–17]. Moreover, associations between the HLA-DRB1 shared epitope (SE) alleles, PTPN22 gene polymorphisms and smoking have been found in ACPA^+^ patients [4, 18–21].

The association between HLA-DRB1 SE and ACPA positivity implicates a role for MHC class II-dependent CD4^+^ T cell activation in ACPA^+^ RA [22]. In line with this, ACPAs are of the IgG subtype, which indicates that ACPA-producing B cells have undergone T cell-dependent class switching [23]. Moreover, citrullinated epitope specific T cells have been identified in ACPA^+^ patients [24–26].

CD4^+^ T helper (Th) cells and their cytokines play a central role in RA pathogenesis [27]. In early RA, pro-inflammatory T cells migrate to inflammatory sites and contribute to disease progression [27–29]. Cytokines produced by T cells, such as TNFα and IL-17A, are involved in activation of local cells and in inflammatory cell recruitment [29–31].

Th cell populations are characterized by differential chemokine receptor expression. For instance, IFNγ producing Th1 cells are CCR6^−^CXCR3^+^CCR4^−^, and IL-4 producing Th2 cells are CCR6^−^CXCR3^−^CCR4^+^ [32]. IL-17A and IL-22 producing cells are primarily found in the heterogeneous CCR6^+^ T cell population, with its subpopulations based on CXCR3, CCR4 and CCR10 expression. CCR6^+^ cells with Th17 characteristics are CXCR3^−^CCR4^+^CCR10^−^ and CCR6^+^ cells with Th22 characteristics are CXCR3^−^CCR4^+^CCR10^+^ [33–35]. CCR6^+^CXCR3^+^CCR4^−^ T cells exhibit both Th17 and Th1 features and are named non-classic Th1 or Th17.1 cells [36–38].

Recently we identified a potential role for CCR6^+^ Th cells in the pathogenesis of RA. In particular CCR6^+^ Th cells and not CCR6^−^ Th cells were potent inducers of synovial fibroblast activation. This resulted in a pro-inflammatory feedback loop leading to the induction of pro-inflammatory mediators, such as IL-1β, IL-6 and PGE_2_ and the tissue degrading enzymes MMP-1 and MMP-3. This loop was dependent on TNFα and IL-17A and may play an important role in the progression of an early inflammation towards a chronic persistent arthritis [30, 31].

The strong indications of T cell involvement in ACPA^+^ RA, and the clinical and molecular differences between ACPA^+^ and ACPA^−^ disease, prompted us to investigate differences in Th cell populations between ACPA^+^ and ACPA^−^ RA patients. In this report we describe that ACPA^+^ patients differ from ACPA^−^ patients by significantly higher memory CCR6^+^ Th cell proportions. These findings suggest that pathogenic memory CCR6^+^ Th cells may be involved in the worse disease course observed in ACPA^+^ RA patients.

## Methods

### Patients

We performed a nested matched case–control study including 27 ACPA^+^ and 27 ACPA^−^ treatment-naive early RA patients matched for disease activity score in 44 joints (DAS44), presence of rheumatoid factor (RF), sex, age, duration of complaints and presence of erosions. All patients met the American College of Rheumatology 2010 revised criteria for RA. None had been taking disease modifying anti-rheumatic drugs. Baseline characteristics on which was matched did not significantly differ between groups. Patients were not matched on tender joint count, swollen joint count, C-reactive protein levels (CRP), erythrocyte sedimentation rate (ESR) and titers of RF. Swollen joint count and titers of RF were significantly different between ACPA^+^ and ACPA^−^ patients (Table 1).

**Table 1:** Baseline characteristics of ACPA^+^ and ACPA^−^ treatment-naive patients with early RA

Characteristic	ACPA^+^ patients (n = 27)	ACPA^−^ patients (n = 27)	p value
DAS44 score (mean ± SD)	3.12 ± 0.82	3.14 ± 0.96	0.89
RF positive n (%)	21 (78)	16 (59)	0.07
Sex (female/male)	19/8	18/9	1.0
Age (mean ± SD, years)	48.2 ± 13.4	51.8 ± 13.8	0.31
Duration of complaints (mean ± SD, days)	159.9 ± 87.2	179.1 ± 103.5	0.29
Erosions n (%)	5 (19)	5 (19)	1.0
Tender joint count	9.93 ± 7.23	9.00 ± 7.90	0.30
Swollen joint count	6.63 ± 5.31	9.44 ± 6.67	0.02
ESR (mm/hr)	27.6 ± 22.5	26.3 ± 24.1	0.49
CRP (mg/l)	13.3 ± 30.3	11.1 ± 9.93	0.57
RF titer (IU/ml)	163 ± 327	64.3 ± 157	0.01

This study was embedded in the Treatment in the Rotterdam Early Arthritis Cohort Study (tREACH) and approved by the Medical Ethics Review Board of Erasmus MC Rotterdam. Written informed consent from all patients participating in this study was obtained.

### Flow cytometry and cell culture

Monoclonal antibody stainings, transcription factor detection and flow cytometry were performed as described previously [35]. Fluorochrome labeled antibodies were purchased from eBioscience (San Diego, CA), BD Biosciences, BioLegend (San Diego, CA) and R&D systems (Minneapolis, MN). Fixable Viability Dye and FOXP3 staining buffer sets were purchased from eBioscience. Samples were acquired on a LSRFortessa flow cytometer (BD Biosciences) and analyzed using FlowJo v7.6 research software (Tree Star Inc. Ashland, OR). Cells were gated on the lymphocyte fraction. Th cell populations were sorted with a FACSAria cell sorter (BD Biosciences). Purity of the obtained Th cell populations was ≥ 98 %. Sorted Th cell populations were stimulated with 0.3 μg/ml soluble αCD3 and 0.4 μg/ml αCD28 (Sanquin, Amsterdam, The Netherlands) and cultured for 4 days as described previously [31].

### Quantitative real-time PCR analysis

RNA extraction and cDNA synthesis were performed as described previously [39]. Primers were designed with Probe Finder software and probes were used from the universal probe library (Roche Applied Science, Indianapolis, IN). Quantitative real-time PCR (RT-PCR) was performed and analyzed using the ViiA7 sequence detection system and software (Life Technologies, Carlsbad, CA). Hypoxanthine–guanine phosphoribosyltransferase (HPRT) was used to normalize gene transcription. The following primers and probes (forward, reverse, probe no.) were used: HPRT (5’- tgaccttgatttattttgcatacc-3’, 5’-cgagcaagacgttcagtcc-3’, 73), IL-17A (5’-tgggaagacctcattggtgt-3’, 5’-ggatttcgtgggattgtgat-3’, 8), IFNγ (5’-ggcattttgaagaattggaaag-3’, 5’-tttggatgctctggtcatctt-3’, 21), RORC (5’-cagcgctccaacatcttct-3’, 5’- ccacatctcccacatggact-3’, 69), and TBX21 (5’-tgtggtccaagtttaatcagca-3’, 5’-tgacaggaatgggaacatcc-3’, 9).

### Statistical analysis

Differences between experimental groups were tested with Wilcoxon matched-pairs signed-ranks test using Prism software v5.04 (GraphPad Software Inc. La Jolla, CA), unless otherwise indicated. *P*-values <0.05 were considered significant.

## Results

### Elevated proportions of CCR6^+^ and CXCR3^+^ Th cell subpopulations in ACPA^+^ patients with early RA

**Fig. 1:**
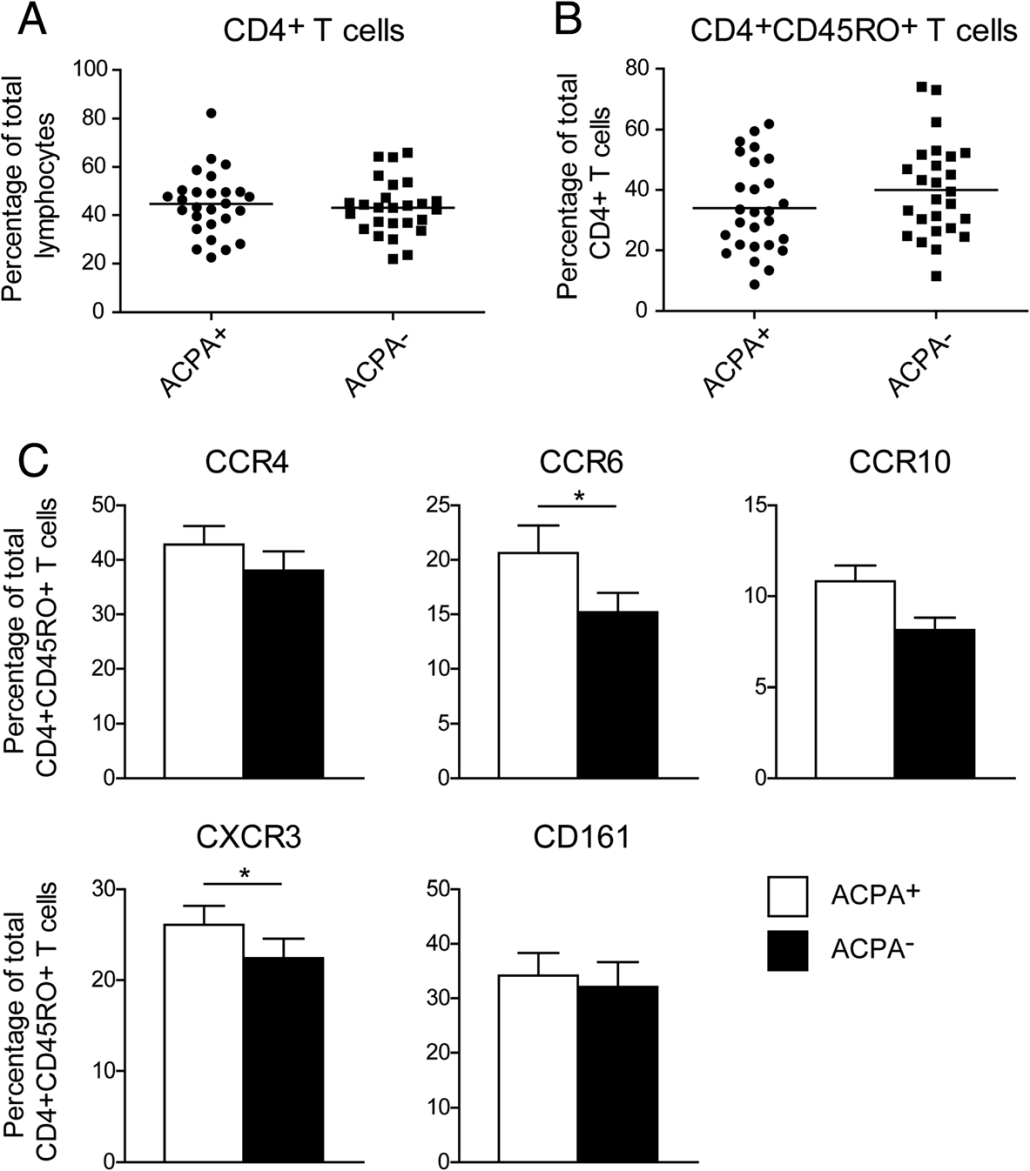
Proportions of chemokine receptor expressing memory Th cells differ between ACPA^+^ and ACPA^−^ RA patients. **a** Fraction of CD4^+^ T cell population within the total lymphocyte population of 27 ACPA^+^ and 27 ACPA^−^ patients with RA. **b** Fraction of memory CD4^+^ T cell population within the total CD4^+^ T cell population of 27 ACPA^+^ and 27 ACPA^−^ patients with RA. **c** Chemokine receptor and CD161 expression on peripheral blood memory (CD45RO^+^) CD4^+^CD25^−^ T cells from matched ACPA^+^ and ACPA^−^ patients with RA, measured by flow cytometry. For statistical analysis Wilcoxon matched-pairs signed-ranks test was performed (* = *p* < 0.05).

In a previous study we found that the proportion of CD4^+^CD45RO^+^ (memory) T cells in PBMC of treatment-naive early RA patients was higher than that in healthy controls [31]. Therefore, we first checked for differences in the total CD4^+^ T cell or the memory CD4^+^ T cell populations between the 27 matched ACPA^+^ and ACPA^−^ subjects. Flow cytometry showed similar proportions of both populations (Fig. 1a and b).

Memory CD4^+^ T cell populations can be characterized by differential expression of the chemokine receptors CCR4, CCR6, CCR10 and CXCR3 and the surface receptor CD161 [32–34, 36]. Receptor expression analysis revealed significantly higher proportions of CCR6 and CXCR3 expressing memory CD4^+^ T cells in ACPA^+^ patients compared to ACPA^−^ patients. No significant differences were found for Th cells expressing CCR4, CCR10 or CD161 (Fig. 1c).

### Elevated proportions of Th22, Th17.1 and unclassified CCR6^+^ Th cells in ACPA^+^ patients with early RA

**Fig. 2:**
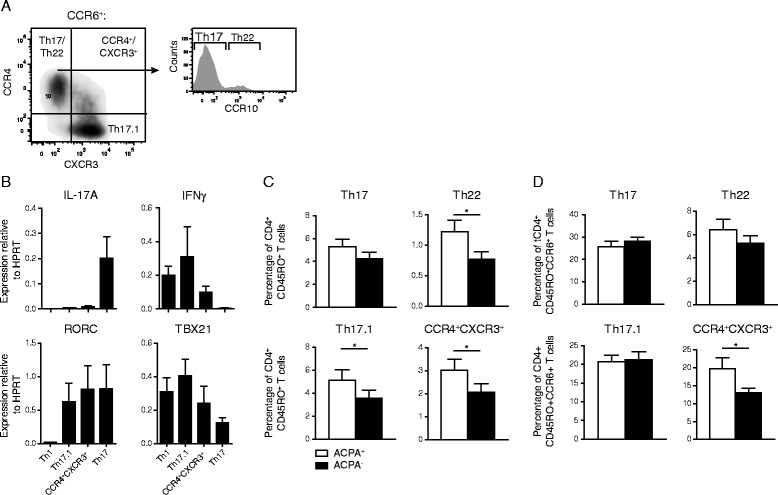
Memory CCR6^+^CD4^+^ T cell subpopulations are increased in ACPA^+^ patients compared to matched ACPA^−^ patients. **a** Gating strategy for the identification of peripheral blood Th17, Th17.1, Th22 and CCR4/CXCR3 DP cell subpopulations. CCR6^+^ cells were gated on CD4^+^CD45RO^+^CD25^−^ T cells. **b** Real-time PCR expression analysis for IL-17, IFNγ, RORC and TBX21 in sorted Th1, Th17, Th17.1 and CCR4/CXCR3 DP CCR6^+^ Th cells obtained from patients with RA (8–10 patients per population). Prior to RNA isolation cells were stimulated with antiCD3/CD28 and cultured for 3 days. **c-d** Proportions of the indicated CD4^+^ T cell subpopulations within the total memory CD4^+^ T cell population (**c**) and memory CCR6^+^CD4^+^ T cell population (**d**) of 27 ACPA^+^ and 27 ACPA^−^ patients with RA. For statistical analysis Wilcoxon matched-pairs signed-ranks test was performed (* = *p* < 0.05).

T cells co-express different chemokine receptors on their surface. Specific combinations of CCR4, CCR6, CCR10 and CXCR3 are expressed by human Th cell populations. We applied a chemokine receptor gating strategy to identify memory CD4^+^ T cells with a Th1, Th2, Th17, Th22 or a Th17.1 profile. Within the CD4^+^CD45RO^+^CD25^−^ T cell population, cells positive for CCR6 expression were gated. Within this CCR6^+^ population, Th17 cells were gated as CXCR3^−^CCR4^+^CCR10^−^ and Th22 cells as CXCR3^−^CCR4^+^CCR10^+^ [33–35]. Th17.1 cells were gated as CXCR3^+^CCR4^−^. Using this gating strategy (Fig. 2A), an unclassified subpopulation was identified, that was double-positive (DP) for the expression of CCR4 and CXCR3. Recently we validated the gating strategy for Th17 and Th22 cells of patients with RA [35]. To validate the gating strategy for the other CCR6^+^ subpopulations we sorted Th1, Th17, Th17.1 and CCR4/CXCR3 DP CCR6^+^ Th cells from patients with RA and analyzed their Th17 and Th1 profile by the transcription levels of IL-17A, IFN-γ, RORC and TBX21. These analyses confirmed the expression profile of Th1, Th17 and Th17.1 as reported previously [36–38]. The CCR4/CXCR3 CCR6^+^ DP were IL-17A low and RORC^+^ with intermediate IFN-γ and TBX21 levels (Fig. 2b). This gating strategy was applied to PBMCs of ACPA^+^ and ACPA^−^ early RA patients. Proportions of the CCR6^+^ Th cell subpopulations Th22, Th17.1 and CCR4/CXCR3 DP Th cells were significantly higher in ACPA^+^ than in ACPA^−^ patients. No statistical significant (p = 0.10) difference was reached for the distribution of Th17 cells between ACPA^+^ and ACPA^−^ patients (Fig. 2c).

To investigate whether the observed increases in Th22, Th17.1 and CCR4/CXCR3 DP memory Th cell populations in ACPA^+^ were due to the overall increase in CCR6^+^ Th cells in these patients (Fig. 1c) or that specific increases were taking place, the CCR6^+^ Th cell populations were expressed as proportion of total CCR6^+^ Th cells. Interestingly, the proportions of all CCR6^+^ subpopulations were comparable between ACPA^+^ and ACPA^−^ patients, except the proportion of CCR4/CXCR3 DP Th cells, that was significantly higher in ACPA^+^ patients than ACPA^−^ patients (Fig. 2d).

These findings show that, in ACPA^+^ patients, proportions of Th22, Th17.1 and CCR4/CXCR3 DP subpopulations were significantly larger than in ACPA^−^ patients. These increases are mainly attributed to the observed increase in the proportion of total CCR6^+^ Th cells.

### Th1 and Th2 proportions are similar in ACPA^+^ and ACPA^−^ patients with RA, but the CCR4/CXCR3 DP CCR6^−^ Th cell subpopulation is elevated in ACPA^+^ patients

**Fig. 3:**
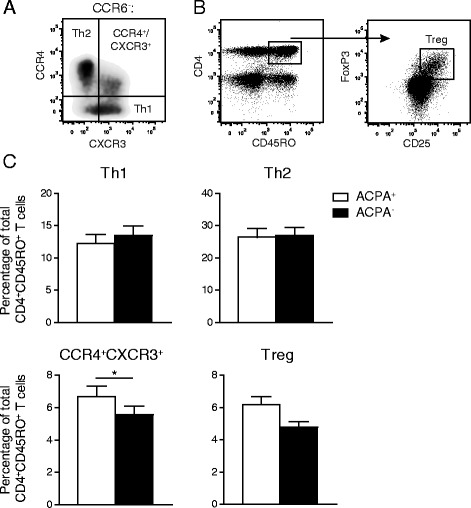
Differences in CCR6^−^CD4^+^ T cell subpopulations and Tregs between ACPA^+^ patients and matched ACPA^−^ patients. **a** Gating strategy for the identification of peripheral blood Th1, Th2 and CCR4/CXCR3 DP cell subpopulations. CCR6^−^ cells were gated on CD4^+^CD45RO^+^CD25^−^ T cells. **b** Gating strategy to identify Treg (CD25^hi^FOXP3^+^) cells within the memory CD4^+^ T cell population. Cells were gated on the total lymphocyte population. **c** Proportions of the indicated CD4^+^ T cell subpopulations within the total memory CD4^+^ T cell population of 27 ACPA^+^ and 27 ACPA^−^ patients with RA. For statistical analysis Wilcoxon matched-pairs signed-ranks test was performed (* = *p* < 0.05).

Similar as described above, CCR6^−^ Th cells were gated within the CD4^+^CD45RO^+^CD25^−^ T cell population. Cells with a Th1 and Th2 profile were gated as CXCR3^+^CCR4^−^ and CXCR3^−^CCR4^+^ respectively [32]. CCR4/CXCR3 DP cells were identified in the CCR6^−^ Th cell fraction (Fig. 3a). The combination of FOXP3 and high CD25 expression by memory CD4^+^ T cells was used to identify T regulatory (Treg) cells (Fig. 3b). Proportions of Th1 and Th2 cells did not differ between the groups. In contrast, the CCR4/CXCR3 DP CCR6- Th cell subpopulation was higher in ACPA^+^ patients than in ACPA^−^ patients (Fig. 3c). Moreover, we found a trend for larger Treg proportions in ACPA^+^ patients than in ACPA^−^ patients (p = 0.06).

### Disease duration correlates with CCR6^+^ Th cell proportions in ACPA^−^, but not ACPA^+^ patients

**Fig. 4:**
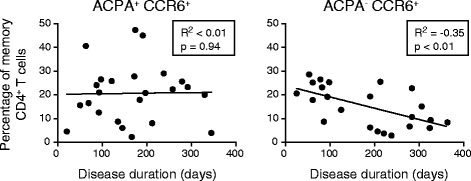
Inverse correlation between proportions of CCR6^+^CD4^+^ T cell subpopulations and disease duration in ACPA^−^ patients. Correlation of the percentage of CCR6^+^CD4^+^ cells (as percentage of total memory CD4^+^ T cells) with self-reported disease duration in ACPA^+^ and ACPA^−^ patients with RA. Pearson correlation test was used to calculate the correlation coefficients (R^2^) and p-values.

ACPA^+^ and ACPA^−^ patients with RA have a similar clinical presentation in the very early phase of disease [40], but ACPA^+^ RA is associated with a more severe disease course and erosions [8–13, 41]. Therefore, we investigated whether the differing CCR6^+^ Th cell, CCR6^−^ Th cell and Treg proportions between ACPA^+^ and ACPA^−^ patients were associated with patient-reported disease duration. Disease duration was not associated with CCR6^+^ Th cell proportions in ACPA^+^ patients, whereas it was significantly inversely correlated with CCR6^+^ Th cell proportions in ACPA^−^ patients (Fig. 4). Additionally, disease duration was significantly positively correlated with CCR6^−^ Th cell proportions in ACPA^−^, but not in ACPA^+^ patients (Table 2). In contrast, neither in ACPA^+^ nor in ACPA^−^ patients disease duration was associated with Treg proportion. Further analysis of the CCR6^+^ Th cell compartment in ACPA^−^ patients showed that Th17 cells and CCR4/CXCR3 DP CCR6^+^ Th cells had a significant inverse correlation with the disease duration. Within the CCR6^−^ Th cell subpopulations, no significant correlations were found (Table 2). In addition, we found a small but significant inverse correlation between the disease duration with the DAS in all patients together (R^2^ = −0.07, p < 0.05), but not in ACPA^+^ patients only (R^2^ = −0.13, p = 0.06) and ACPA^−^ patients only (R^2^ = −0.04, p = 0.31).

**Table 2:** Correlation of Th cell populations (% memory Th cells) with disease duration

	ACPA^+^ patients		ACPA^−^ patients	
Population	R^2^	P value	R^2^	P value
CCR6^+^ total	<0.01	0.94	−0.35	<0.01
Th17.1	0.02	0.50	−0.13	0.09
Th22	−0.04	0.37	−0.10	0.14
Th17	<0.01	0.91	−0.27	0.01
CCR4/CXCR3 DP CCR6^+^	<0.01	0.69	−0.36	<0.01
Treg	<0.01	0.76	0.04	0.31
CCR6^−^ total	<−0.01	0.82	0.37	<0.01
Th1	0.09	0.14	<0.01	0.79
Th2	<−0.01	0.99	0.03	0.43
CCR4/CXCR3 DP CCR6^−^	0.02	0.51	−0.11	0.13

These data show that CCR6^+^ Th cell subpopulations are inversely correlated with disease duration in ACPA^−^ patients but not in ACPA^+^ patients.

## Discussion

In the present study we found that ACPA^+^ patients have a higher proportion of peripheral CCR6^+^ Th cells than ACPA^−^ patients. Chemokine receptor expression analysis revealed an increase in Th22, Th17.1 and CCR4/CXCR3 DP CCR6^+^ Th cells. These increases are mainly attributed to the observed increase in the proportion of total CCR6^+^ Th cells. In addition, ACPA^+^ patients had higher proportions of CCR4/CXCR3 DP CCR6^−^ Th cells than ACPA^−^ patients, but did not differ in Th1 and Th2 proportions. We also found increases in Th17 and Treg proportions in ACPA^+^ patients, but these differences did not reach statistical significance. Furthermore, in ACPA^+^ patients the proportion of CCR6^+^ Th cells was not correlated to disease duration, whereas in ACPA^−^ patients proportions of CCR6^+^ Th cells were negatively correlated with increasing disease duration.

Our findings that the distribution of Th cell populations is dependent on ACPA status is in line with previous studies linking ACPA^+^ with the CD4^+^ T cell component: (i) There is a strong association between ACPA positivity and MHC class II-restricted HLA-DRB1 SE alleles; (ii) ACPAs are mainly of the IgG subtype, which are normally synthesized after T cell-mediated immunoglobulin loci class switching; (iii) citrullinated peptide specific CD4^+^ T cells are present in ACPA^+^ patients [23–26]. Furthermore, PTPN22 gene polymorphisms may be involved in the formation of citrullinated peptide-specific CD4^+^ T cells and therefore be a risk factor for ACPA^+^ RA [42].

The identification of increased CCR6^+^ Th populations in ACPA^+^ RA suggest that these cells are implicated in the more severe disease course of patients with ACPA^+^ RA. In this context, we recently identified a potential pathogenic role for CCR6^+^ Th cells obtained from treatment naive patients with early RA. This included the role of CCR6^+^ Th cells as potent inducers of a pro-inflammatory loop, driven by autocrine IL-17A production and resulting in the induction of IL-1β, IL-6, IL-8, PGE_2_ and MMPs by synovial fibroblasts [31, 35]. This may suggest that CCR6^+^ Th cells are involved in the amplification of inflammatory reactions resulting in the more severe disease course observed in ACPA^+^ RA.

Given this pathogenic role of CCR6^+^ Th cells, it is of particular interest that we found no correlation between the proportion of CCR6^+^ Th cells and disease duration in ACPA^+^ patients, while in ACPA^−^ patients proportions of CCR6^+^ Th cells were negatively correlated with increasing disease duration. However, the time of onset of disease is self-reported, and therefore an estimation. Additionally, it is possible that the worse disease course in ACPA^+^ patients might lead to earlier recognition of disease onset by ACPA^+^ patients than by ACPA^−^ patients, skewing the estimated time of onset [8–10]. Nevertheless, these data might suggest that CCR6^+^ Th cells are involved in the maintenance of inflammation in ACPA^+^ RA and may underlie the differences in treatment outcome between ACPA^+^ and ACPA^−^ RA.

In a previous study it was found that IL-17A responses by CD4^+^ T cells of ACPA^+^ patients with RA were induced after culture with citrullinated peptides. This was not the case for PBMCs from healthy controls. In addition, the level of IL-17A production correlated strongly with the level of proliferation in response to citrullinated peptides [26]. This indicates that IL-17 producing T cells, and therefore CCR6^+^ Th cells as well, might be particularly important in responses to citrullinated protein. Interestingly, it has been suggested that a SNP in the CCR6 gene, which is associated with RA, is more strongly linked to ACPA^+^ RA than ACPA^−^ RA [43–45]. Future research should clarify whether CCR6^+^ Th cells have T cell receptors specific for citrullinated peptides. Alternatively, it would be interesting to compare CCL20 levels in synovial fluid of ACPA^+^ and ACPA^−^ patients, since CCL20 is the only known ligand for CCR6 [46, 47]. Higher CCL20 levels in synovial fluid of ACPA^+^ patients could also account for higher CCR6^+^ Th cell numbers.

The CCR6^+^ Th population is heterogeneous, and based on differential chemokine receptor expression various subpopulations can be identified. The roles and contribution of these CCR6^+^ Th populations in the severity of ACPA^+^ RA are unclear. Recently we found that Th22 cells were not required for Th17/IL-17 mediated synovial inflammation [35]. On the other hand, Th22 cells were shown to be associated with erosive disease and serum IL-22 levels correlate with serum ACPA titers [48, 49]. Moreover IL-22 was able to promote osteoclastogenesis by inducing RANKL in synovial fibroblasts [49].

The ontogeny of the Th17.1 CCR6^+^ Th cell subpopulation is unclear. Th17.1 cells might be derived from Th17 cells, as culturing of human Th17 cells in the presence of IL-12 up-regulates Th1 characteristics like TBX21 and IFNγ and down-regulates Th17 characteristics like RORC and IL-17, leading to a Th17.1-like phenotype [50–52]. On the other hand, Th17.1 cells may also originate directly from human naive T cells. Upon interaction with *Candida albicans* primed monocytes, naive T cells develop into cells with Th17.1 characteristics [53]. Recently, particular Th17.1 cells were found to have a pathogenic signature, specifically those that expressed the transporter protein multi-drug resistance type 1 (MDR1), and thereby became unresponsive to glucocorticoids [37]. The pathogenic signature and drug-resistance suggest the clinical importance of Th17.1 cells in RA. The origin and development of the CCR4/CXCR3 CCR6^+^ and CCR6^−^ Th subpopulations are also ill-defined, and these populations might resemble intermediate or transitional Th cells. Research to factors that foster the development of these cells is lacking, but one possibility is that the micro-environment, such as concentrations of the cytokines IL-12, IL-23 and IL-6 that are important in Th1 and Th17 differentiation, plays a role. More research is needed to investigate the ontogeny, stability, characteristics and functions of these subpopulations.

Surprisingly, we found higher Treg proportions in ACPA^+^ patients, although the difference did not reach statistical significance (p = 0.06). Tregs normally play an immune suppressive role. It might be that these increased Tregs are induced as a feedback mechanism to control the increased proportions of CCR6^+^ Th cells. However, Tregs are able to convert to Th17 cells [54, 55]. Especially these converted cells are key to the development of autoimmune arthritis [56]. Future research should point out whether the Tregs in (ACPA^+^) RA patients are functional and could convert to Th17 cells.

## Conclusions

In this study we have found that Th cell distributions are associated with ACPA status. In particular CCR6^+^ Th cell proportions were higher in ACPA^+^ RA in comparison to ACPA^−^ RA. Moreover, CCR6^+^ Th cells are inversely correlated with disease duration in ACPA^−^ patients but not in ACPA^+^ patients. These findings point toward a pathogenic role for CCR6^+^ Th cells in the more severe disease course of patients with ACPA^+^ RA and imply a role for CCR6^+^ Th cells in the differences observed in the treatment outcome of patients with ACPA^+^ and ACPA^−^ RA.

## Abbreviations

ACPA: anti-citrullinated protein antibodies
CCR: C-C chemokine receptor
CD: cluster of differentiation
CXCR3: CXC chemokine receptor 3
DAS44: disease activity score, 44 joints evaluated
DP: double-positive
FOXP3: forkhead box P3
HLA: human leukocyte antigen
IFNγ: interferon gamma, IgG, immunoglobulin G
IL: interleukin
MDR: multi-drug resistance type 1
MHC: major histocompatibility complex
MMP: matrix metalloproteinase
PBMCs: peripheral blood mononuclear cells
PGE_2_: prostaglandin E_2_
PTPN22: Protein tyrosine phosphatase, non-receptor type 22
RA: Rheumatoid arthritis
RANKL: receptor activator of nuclear factor kappa-B ligand
RF: Rheumatoid factor
SD: Standard deviation
SE: shared epitope
Th: T helper
TNFα: tumor necrosis factor alpha
Treg: regulatory T cell
